# T3_MM: A Markov Model Effectively Classifies Bacterial Type III Secretion Signals

**DOI:** 10.1371/journal.pone.0058173

**Published:** 2013-03-05

**Authors:** Yejun Wang, Ming'an Sun, Hongxia Bao, Aaron P. White

**Affiliations:** 1 Genomics Research Center, Haerbin Medical University, Harbin, China; 2 School of Life Science, the Chinese University of Hong Kong, Shatin, New Territories, Hong Kong; 3 Vaccine and Infectious Disease Organization, University of Saskatchewan, Saskatoon, Saskatchewan, Canada; University of Nottingham, United Kingdom

## Abstract

**Motivation:**

Type III Secretion Systems (T3SSs) play important roles in the interaction between gram-negative bacteria and their hosts. T3SSs function by translocating a group of bacterial effector proteins into the host cytoplasm. The details of specific type III secretion process are yet to be clarified. This research focused on comparing the amino acid composition within the N-terminal 100 amino acids from type III secretion (T3S) signal sequences or non-T3S proteins, specifically whether each residue exerts a constraint on residues found in adjacent positions. We used these comparisons to set up a statistic model to quantitatively model and effectively distinguish T3S effectors.

**Results:**

In this study, the amino acid composition (Aac) probability profiles conditional on its sequentially preceding position and corresponding amino acids were compared between N-terminal sequences of T3S and non-T3S proteins. The profiles are generally different. A Markov model, namely T3_MM, was consequently designed to calculate the total Aac conditional probability difference, i.e., the likelihood ratio of a sequence being a T3S or a non-T3S protein. With T3_MM, known T3S and non-T3S proteins were found to well approximate two distinct normal distributions. The model could distinguish validated T3S and non-T3S proteins with a 5-fold cross-validation sensitivity of 83.9% at a specificity of 90.3%. T3_MM was also shown to be more robust, accurate, simple, and statistically quantitative, when compared with other T3S protein prediction models. The high effectiveness of T3_MM also indicated the overall Aac difference between N-termini of T3S and non-T3S proteins, and the constraint of Aac exerted by its preceding position and corresponding Aac.

**Availability:**

An R package for T3_MM is freely downloadable from: http://biocomputer.bio.cuhk.edu.hk/softwares/T3_MM. T3_MM web server: http://biocomputer.bio.cuhk.edu.hk/T3DB/T3_MM.php.

## Introduction

The bacterial type III secretion system (T3SS) is a needle-like hollow secretion apparatus which is expressed on the surface of a wide variety of gram-negative bacteria [1]–[4]. T3SS can mediate the translocation of a group of proteins from bacteria into the cytoplasm of host cells; therefore, it plays an important role in the interactions between bacteria and their hosts [1], [5]–[6]. The proteins specifically recognized, secreted and translocated by the T3SS are called type III secreted (T3S) effectors, which exert their biological activities in host cells in concert to cause pathogenicity [7].

The N-termini of T3S effectors have been shown to contain important signals that guide their specific recognition by T3SSs [8]–[14]. Due to great diversity, no consensus sequences or common motifs have been identified from this region of T3S effectors [10], [15]–[16]. The limited physicochemical property preference of amino acids (e.g., charged and polar) only gave few clues about the specificity of T3S signals [15]. It was suggested that N-termini of T3S effectors frequently adopted more flexible secondary or tertiary structure [7]. However, integration of these secondary structure features and others (such as solvent accessibility) did not facilitate the identification of T3S signal sequences, indicating that these properties in T3S proteins couldn't be distinct from those in non-T3S proteins, and they couldn't be the major factors guiding specific type III recognition [15], [17]. Till now, very few N-terminal sequences of T3S effectors have been resolved for three-dimensional structures, hindering the observation and inference of the possible recognition specificity of T3S signals. No simple, general and comprehensively-representative features have been observed, which could well distinguish T3S and non-T3S proteins. Consequently, instead, multiple-aspect, subtle, and partially-representative properties of T3S signal sequences were analysed, extracted and combined to train different non-linear classification models [15], [17]–[20]. These models greatly prompted the identification of new effectors, and meanwhile facilitated our understanding of type III secretion mechanisms and the evolution of T3S effectors.

Here, we further explored the features differently represented by T3S and non-T3S proteins. Different amino acids have been observed to be preferred in T3S N-terminal sequences generally [15] and position-specifically [17]. For example, serine is most preferred by T3S signal sequences generally and specifically in most positions [10], [15], [17]. Does this mean that a stretch of serines should be theoretically the best optimal T3S signal sequence? Could amino acids in a T3S signal sequence exert some constraint on amino acid composition (Aac) at their sequentially succeeded positions? Furthermore, is this constraint different from that of non-T3S sequences? If so, could these features be used for discriminating T3S proteins? This study aimed to answer these questions.

## Results

### Different Aac Probability Profiles Conditional on Amino Acids at Sequentially Preceding Position

We previously reported that specific type III secretion guiding signals were buried in the N-terminal 100 amino acid region of T3S effectors [17]. In this study, N-terminal 100 amino acids of T3S and non-T3S proteins were extracted for feature analysis. The absolute composition of each species of amino acid was calculated and compared between T3S and non-T3S sequences. Furthermore, to observe the constraint of specific amino acids on the Aac at succeeding positions, the conditional probability of each amino acid on each amino acid at the preceding position was also calculated and compared.

As for absolute composition of single amino acids, T3S sequences had higher proportion of serine, leucine and alanine and lower proportion of tryptophan, cysteine, tyrosine and methionine (Fig. S1). However, T3S and non-T3S sequences didn't show marked differences except for serine and tryptophan, with serine being enriched and tryptophan being depleted in T3S sequences (Fig. S1).

**Figure 1:**
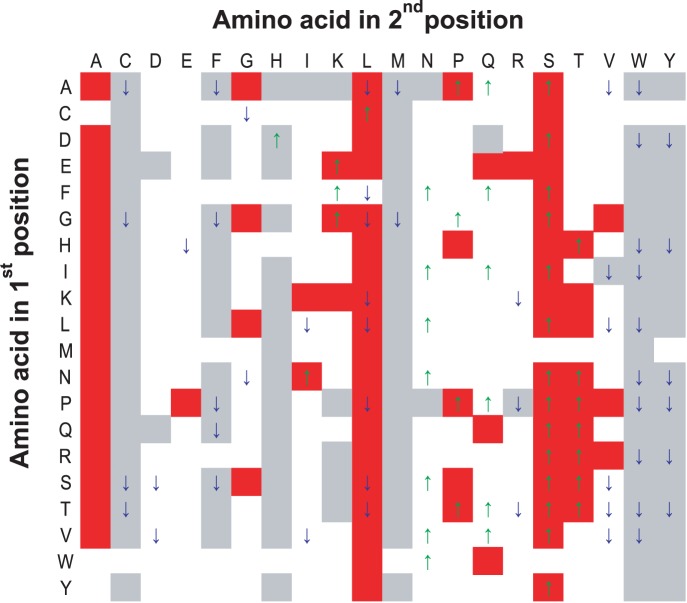
Distribution of bi-amino acids (bi-aa) with significant difference of Aac conditional probability in T3S signal sequences. The vertical and horizontal axis represents the 1^st^ and 2^nd^ amino acid respectively. A binomial distribution-based statistic test was performed to each amino acid at the second position given the first amino acid. The second amino acid with significantly biased composition compared with theoretical random distribution was highlighted in red (enriched) or grey (depleted) background. The second amino acid with significantly biased composition compared with non-T3S sequences was indicated with an upward (higher in T3S sequences) or downward (depleted in T3S sequences) arrow. Benjamini & Hochberg correction for multiple tests was adopted to control the type I errors [30]. The False Discovery Rate (FDR) was set as ≤0.05.

For each amino acid, the probability conditional on each type of preceding amino acid was compared with its absolute probability. As shown in Fig. S2 (marked with star above the bar), there were many amino acids for which the conditional probability was significantly different from their absolute probability in T3S sequences (p<0.05), indicating probabilistic dependence of Aac on its sequentially preceding amino acid. The difference was not significant in non-T3S sequences (data not shown), indicating there was a T3S-specific Aac dependence or constraint. An alternative binomial distribution-based statistics was also adopted to compare the real conditional probability of each amino acid with its expected value under independent hypothesis. The results further confirmed the conclusion about position dependency in T3S sequences (Fig. 1; red and grey background representing significantly enriched and depleted amino acids in real T3S sequence, respectively; significance set as FDR <0.05). The T3S-specific Aac dependence was tested further with the third strategy. On each amino acid, the conditional probability of its succeeding amino acid was ordered and ranked. The rank difference between the conditional and the absolute probability of each type of succeeding amino acid was calculated. There were a lot of cases, which were observed with significant rank difference (absolute value of difference ≥5; Fig. S2, marked with upward or downward arrow above the bar). The results further confirmed that the Aac in T3S sequences was influenced by its preceding position.

Many Aac conditional probabilities on each amino acid at preceding position were significantly different between T3S and non-T3S sequences (Fig. 1, with upward or downward arrow; significance set as FDR <0.05). Notably, the proportion of leucine was generally lower in T3S sequences than in non-T3S sequences, though it was frequently enriched after different types of amino acids in T3S sequences (Fig. 1). Similarly, alanine was no longer differentially composed between T3S and non-T3S sequences; cysteine, histidine and methionine were not strikingly depleted in T3S sequences compared with non-T3S sequences (Fig. 1). Some other amino acids, e.g., aspartic acid and glutamine, were frequently higher while valine was frequently lower in T3S than non-T3S sequences, though they were not significantly enriched or depleted in T3S sequences (Fig. 1).

The ranks of Aac conditional probabilities on each amino acid at preceding position also showed an apparent difference between T3S and non-T3S sequences (Fig. S2; marked with backward or forward arrow below the bar). This difference could be caused by the superimposed or loosened constraint by adjacent position in T3S proteins (or in non-T3S proteins). For example, when the first position is isoleucine, the ranks of asparagine and valine were apparently different in T3S and non-T3S sequences (Fig. S2). The higher and lower order of asparagine and valine composition in T3S than non-T3S sequences, was potentially caused by the increased and decreased asparagine and valine after isoleucine, respectively, in T3S sequences (Fig. S2). There were many similar examples, such as ‘NI’ and ‘NG’, ‘PP’ and ‘PR’, ‘WN’ and ‘WK’, and so on (Fig. S2). Therefore, there were different Aac probability profiles conditional on amino acids at sequentially preceding position in T3S and non-T3S sequences.

Fig. S2 also demonstrated a trend that serine was preferred after each type of amino acid in the preceding position in T3S sequences. However, statistically, the dimer ‘SS’ was not most significantly enriched when compared to other dimers (Fig. S2). The occurrence rank of ‘SS’ among dimers beginning with ‘S’ was not most significantly different between T3S and non-T3S sequences (Fig. S2). This might partly explain why a continual stretch of serines was not frequently found in N-terminal sequences of T3S proteins.

### Probabilistic Modeling of the Overall Difference of Conditional Probability Profiles between T3S and Non-T3S Proteins

A sequential likelihood ratio variable based on Markov model, *R*, was created to measure the overall difference of conditional probability profiles on position-adjacent Aac between T3S and non-T3S proteins (Methods). The *R* values were calculated and statistically analyzed for T3S and non-T3S sequences.

**Figure 2:**
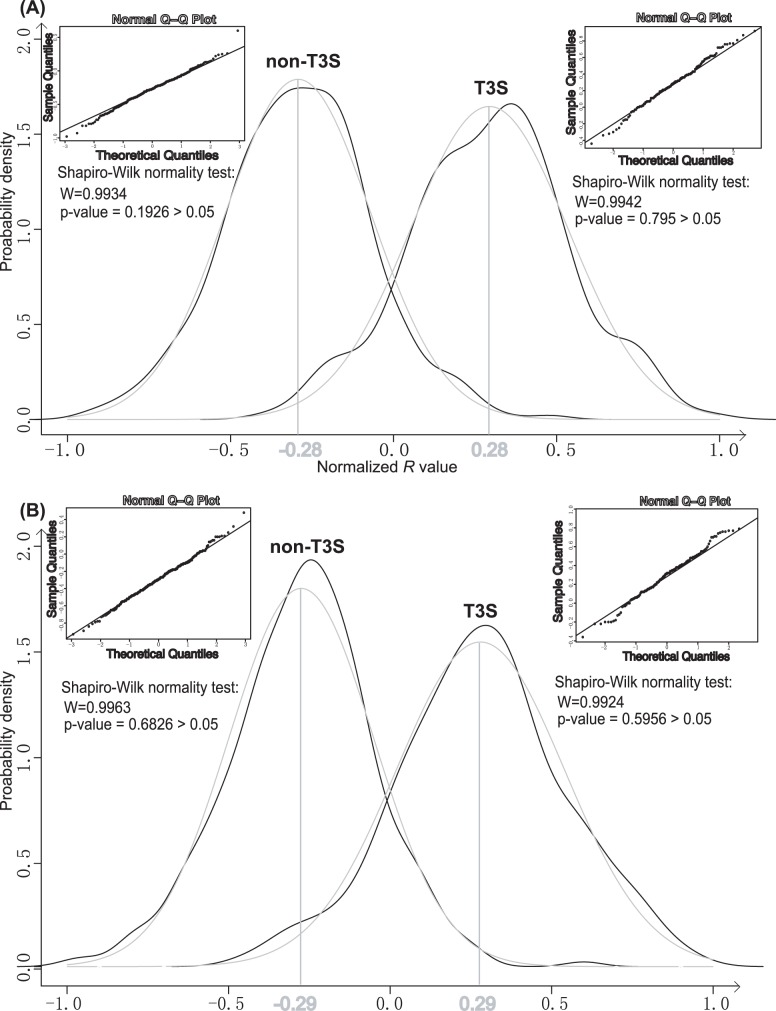
Probabilistically modelling the overall difference of conditional probability profiles of T3S and non-T3S sequences. The distribution (black curves) and normal approximations (grey curves) of T3S and non-T3S *R* values (A) and weighted *R* values (B) were shown. The means of approximated normal distributions were also indicated. For each normal approximation, the Normal Q–Q plot and Shapiro-Wilk normality test results were shown nearby corresponding distribution curve.

As shown in Fig. 2A, the *R* values for T3S and non-T3S sequences could be fit to two distinct distributions. According to the forms, the distribution of T3S *R* values was approximated to a normal distribution with a mean of 0.28 and a standard deviation of 0.26, while the distribution of non-T3S *R* values was approximated to another normal distribution with a mean of −0.28 and a standard deviation of 0.22 (Fig. 2A). Both normal Q–Q plot analysis and Shapiro-Wilk normality test supported the normal approximation for the two distributions (Fig. 2A).

The absolute probabilities of individual amino acids were considered as coefficients to calculate the weighted *R* values of T3S and non-T3S sequences. Like *R* values, the weighted *R* values of two types of proteins also followed distinct approximated normal distribution (mean and standard deviation were respectively 0.29 and 0.24 for T3S sequences, and −0.29 and 0.22 for non-T3S sequences) (Fig. 2B).

### Classification of T3S Signal Sequences Based on *R* and Derived Values

Based on the probabilistic modeling results, a protein could be classified as T3S or non-T3S sequence according to a selected cutoff *R* value. The training dataset with 154 putative T3S effectors and 308 non-T3S proteins were used for classifying performance evaluation (Text S1).

**Figure 3:**
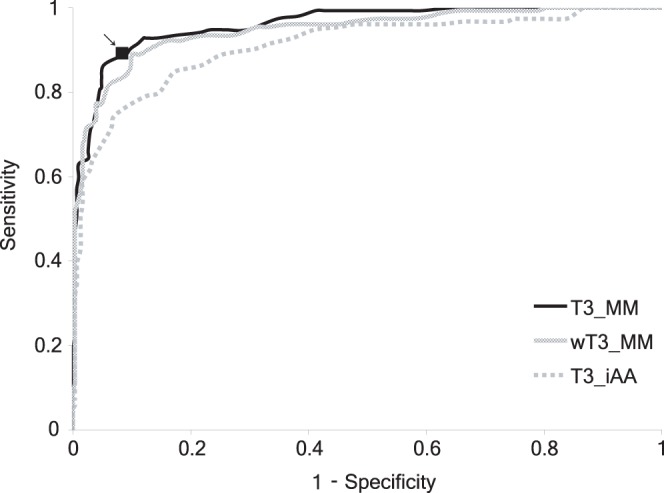
Receiver Operating Characteristic curves of different T3S protein classification models. The point of cutoff value (*R* = 0) was indicated with a black rectangle and an arrow.

With T3_MM, the prediction model based on distribution of *R* values, the sensitivity and selectivity varied with different cutoff values (Fig. 3). According to the ROC curve, an optimized cutoff was selected, ensuring the best distinguishing power. For T3_MM, a cutoff *R* value of 0 could well distinguish T3S sequences, with a sensitivity of 89.6% at a selectivity of 90.9% (Fig. 3 and Table 1). The cutoff based on ROC curve was very similar to the discriminant function resulted value (−0.025, Methods).

**Table 1:** The classifying performance of different models on T3S and non-T3S training data.

Model	Cutoff value	*S_n_* (%) vs. *S_p_* (%)	*A* (%)	MCC
T3_MM	0	89.61 vs. 90.91	90.48	0.7911
wT3_MM	0	87.66 vs. 90.26	89.39	0.7666
T3_iAA	0	79.22 vs. 86.04	83.77	0.6420

The parameters were calculated based on training-reclassifying results for training dataset.

The performance of weighted *R* values based model (wT3_MM) and individual amino acid probability based model (T3_iAA) were also evaluated, and compared with that of T3_MM (Fig. 3 and Table 1). As shown by the ROC figure and different performance parameters, T3_MM was best among all three models, though the difference between T3_MM and wT3_MM was not striking (Fig. 3 and Table 1). Both T3_MM and wT3_MM significantly outperformed T3_iAA, with respect to sensitivity, specificity, accuracy, MCC, and others (Fig. 3 and Table 1).

Support Vector Machine (SVM), Generalized Linear Model (GLM) and RandomForest (RF) models were also used to train the Aac conditional probability features. Among them, SVM could achieve a better specificity but at a loss of sensitivity and accuracy (Table S1). The other two models, however, performed significantly worse than T3_MM (Table S1).

### Performance Evaluation and Comparison of T3_MM and other Established T3S Protein Prediction Methods

To better evaluate the performance of T3_MM to predict T3S signal sequences, a 5-fold cross validation strategy was adopted to the training datasets. As shown in Table 2, the method achieved an average sensitivity of ∼ 83.9% at a specificity of ∼ 90.3%. There are other well-established software programs to predict T3S proteins, among which BPBAac and Effective T3 were reported with best performance [15], [17]. With the same training dataset, all the parameters including sensitivity, specificity, accuracy, and MCC, showed that, T3_MM performed better than Effective T3 (Table 2) and other softwares such as SIEVE, SSE-ACC, etc. (data not shown). However, BPBAac achieved better performance than T3_MM (Table 2).

**Table 2:** Performance comparison between T3_MM, BPBAac and Effective T3 on training dataset.

Software	*S_n_* (%)	*S_p_* (%)	*A* (%)	MCC
T3_MM	83.87 (±5.10)	90.32 (±5.93)	88.17 (±3.10)	0.7362
BPBAac	90.97 (±7.70)	97.42 (±4.05)	95.27 (±2.57)	0.8929
Effective T3	82.53 (±6.69)	86.63 (±5.42)	86.69 (±3.68)	0.6852

The parameters were evaluated based on a 5-fold cross-validation strategy. The standard deviations for *Sn*, *Sp* and *A* were also indicated.

Other new test datasets, of which the sequences were not included in the training dataset, were also collected to further compare the performance of models. A *Ralstonia* T3S and non-T3S protein dataset was tested, from which all the sequences were not included in the training data of T3_MM or Effective T3, and were excluded from the training data of BPBAac model (Methods; Text S2) [21]. As shown in Table 3, T3_MM could most effectively distinguish the T3S and non-T3S proteins, whereas the BPBAac and Effective T3 seemed not very stable, with a quite low recall value even at a comparable (or slightly higher) selectivity.

**Table 3:** Performance comparison between T3_MM, BPBAac and Effective T3 on new datasets.

Dataset	Software	Recall (%)^a^	Selectivity (%)^b^	*A* (%)
Mukaihara2010	T3_MM	32/35 (91.43)	64/70(91.43)	91.43
	BPBAac	21/35 (60.00)	67/70(94.87)	83.81
	Effective T3	20/35(57.14)	70/78(92.86)	80.95
Baltrus 2011	T3_MM	275/291 (94.50)	539/582 (92.61)	93.24
	BPBAac	234/291 (80.41)	558/582 (95.88)	90.72
	Effective T3	223/291 (76.63)	533/582 (91.58)	86.60

Note: ^a^‘Recall’ was adopted here instead of sensitivity to describe the number of validated T3S proteins correctly predicted from the total number of T3S proteins. The recall percentage was noted within parentheses after recall value, which was identical to the sensitivity.

b ‘Selectivity’ was adopted here instead of specificity, to describe the number of non-T3S proteins correctly predicted from the total number of non-T3S proteins. The selectivity percentage was noted within parentheses after selectivity value, which was identical to the specificity.

To avoid overestimating the general prediction performance of T3_MM based on a specific genus or species, another large-scale T3S dataset from different *Pseudomonas* strains was also included for performance evaluation [22]. Again, T3_MM showed the best classification performance, with marked increase of recall value and general prediction accuracy, at a cost of slightly lowered selectivity (Table 3).

**Figure 4:**
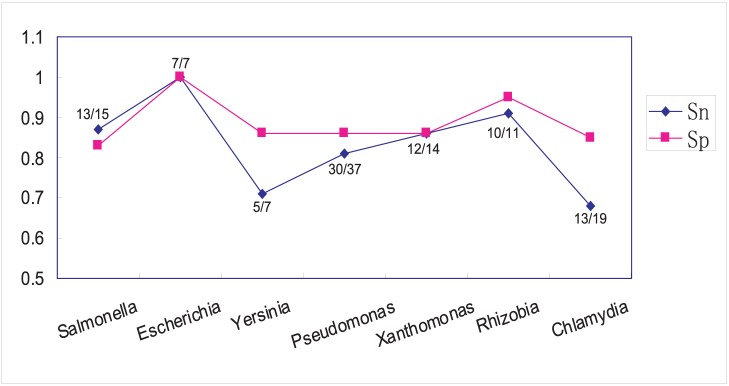
Inter-species cross validation of the T3S effector predictions. The sensitivity (Sn) and specificity (Sp) of classification were shown in blue and purple, respectively. The T3S effector recall of each representative genera or subgroup was also indicated. Genus names are listed below each series of dots.

It is still possible that T3_MM only works well for some specific genera and is not generally applicable. An inter-genera cross validation strategy (Methods) was adopted to observe the inter-genera prediction capability of this *R* value-based model. For most genera, the recall percentage of known effectors was high (∼ 80%) at an acceptable specificity (>85%) (Fig. 4). Even in the worst case scenario (i.e., classification of *Chlamydia* effectors based on non-*Chlamydia* model), 68% of the known effectors (13/19) were correctly recognized (Fig. 4).

Taken together, the T3_MM algortithm was able to efficiently classify T3S proteins from non-T3S proteins with high sensitivity and general prediction accuracy. The model could also be applied to different bacterial genera or phyla. The actual specificity of T3_MM could be higher since the “non-T3S” sequences in the training or testing datasets could contain some unknown effectors.

### Amino Acid Composition Properties of T3S Signal Sequences

Based on the probability matrix and T3_MM model, any given peptide sequence could be calculated for its probability to be a T3S sequence. However, it is extremely computationally demanding to calculate or compare the probability of all possible peptides of length 100 aa. To analyze the preferred and unfavorable amino acid composition in T3S signal sequences, a selected group of continual bi-amino acid (bi-aa) sequences were computationally simulated and classified using T3_MM and BPBAac, respectively (Table S2). According to the general composition of single amino acids and the relative composition preference in T3S and non-T3S proteins (Fig. S1), a sequence purely composed by serine was expected to have the highest prediction value. Consistent with this hypothesis, BPBAac gave the sequence formed by continuous serines the highest score, since the serine composition in most positions was apparently higher than other amino acids and different between T3S and non-T3S proteins (Table S2; Table 4). T3_MM, however, predicted that a string of proline and ‘NS’ (together with ‘SN’ because ‘SN’ could not be excluded from the continual ‘NS’ string) were more likely to be T3S secreted sequences (Table S2; Table 4). The discrepancy between T3_MM and BPBAac results was likely caused by the different basis of the T3_MM model: dependence on sequentially adjacent amino acids rather than absolute or relative composition of individual amino acids. The bi-aa composition of ‘PP’ or ‘NS’ was significantly different between T3S and non-T3S sequences, and the difference was more marked than ‘SS’, leading to the higher T3_MM prediction score for ‘PP’ or ‘NS’ than ‘SS’ string (Fig. 1). Similarly, BPBAac frequently scored bi-aa strings higher values if either of the two amino acids were selectively preferred by T3S proteins, such as ‘SX’ or ‘XS’, where ‘X’ represented any amino acid (Table S2; Table 4). In contrast, T3_MM often gave these bi-aa strings different prediction values. For example, all the ‘SX’ or ‘XS’ strings were predicted with high scores using BPBAac, while the scores predicted by T3_MM were apparently different, with 3 pairs of strings (‘SC’/‘CS’, ‘SW’/’WS’, and ‘SM’/‘MS’) classified as non-T3S sequences (Table S2). Moreover, some amino acids were either not enriched in T3S signal sequences, or not differently preferred by T3S and non-T3S sequences, such as isoleucine and cysteine (Fig. S1). Therefore, most ‘CX’/‘XC’ and ‘IX’/‘XI’ strings (including ‘CI’/‘IC’ string) were predicted by BPBAac to be non-T3S sequences (Table S2). T3_MM also classified strings solely composed of isoleucine or cysteine as non-T3S proteins (Table S2). However, the bi-aa composition of ‘IC’ or ‘CI’ conditional on the preceding amino acid (‘I’ or ‘C’, respectively) was significantly different between T3S and non-T3S proteins, and consequently, T3_MM gave ‘IC’/‘CI’ strings quite high score and classified them to be T3S sequences (Table S2). The 30 bi-aa sequences given the highest and lowest prediction scores with T3_MM and BPBAac, respectively are shown in Table 4.

**Table 4:** The 30 simulated bi-aa sequences of highest (Pos) and lowest (Neg) prediction scores with T3_MM and BPBAac.

T3_MM_Pos	T3_MM_Neg	BPBAac_Pos	BPBAac_Neg
(PP)_50_^a^	(PW|WP)_50_	(SS)_50_	(VV)_50_
(NS|SN)_50_^ b^	(HY|YH)_50_	(PS)_50_	(VD)_50_
(SS)_50_	(DW|WD)_50_	(TS)_50_	(VY)_50_
(NI|IN)_50_	(RC|CR)_50_	(QS)_50_	(VI)_50_
(IC|CI)_50_	(RW|WR)_50_	(RS)_50_	(VG)_50_
(TT)_50_	(GC|CG)_50_	(SP)_50_	(VF)_50_
(ST|TS)_50_	(YY)_50_	(NS)_50_	(FV)_50_
(DH|HD)_50_	(WY|YW)_50_	(ST)_50_	(VK)_50_
(MY|YM)_50_	(WK|KW)_50_	(ES)_50_	(VM)_50_
(CL|LC)_50_	(TC|CT)_50_	(GS)_50_	(FD)_50_
(NH|HN)_50_	(WI|IW)_50_	(IS)_50_	(KV)_50_
(NN)_50_	(HW|WH)_50_	(SN)_50_	(VW)_50_
(QT|TQ)_50_	(WA|AW)_50_	(SR)_50_	(FG)_50_
(AP|PA)_50_	(WF|FW)_50_	(SQ)_50_	(VC)_50_
(NT|TN)_50_	(DY|YD)_50_	(DS)_50_	(KD)_50_
(PS|SP)_50_	(LW|WL)_50_	(LS)_50_	(VE)_50_
(PT|TP)_50_	(KC|CK)_50_	(SE)_50_	(KY)_50_
(IQ|QI)_50_	(MV|VM)_50_	(AS)_50_	(FI)_50_
(HK|KH)_50_	(MC|CM)_50_	(KS)_50_	(FY)_50_
(KF|FK)_50_	(CA|AC)_50_	(SG)_50_	(KI)_50_
(HM|MH)_50_	(DC|CD)_50_	(SA)_50_	(YV)_50_
(QS|SQ)_50_	(VI|IV)_50_	(SL)_50_	(KG)_50_
(AS|SA)_50_	(VY|YV)_50_	(VS)_50_	(FF)_50_
(HH)_50_	(TW|WT)_50_	(SK)_50_	(KF)_50_
(CN|NC)_50_	(QC|CQ)_50_	(SV)_50_	(FK)_50_
(VN|NV)_50_	(YF|FY)_50_	(SD)_50_	(VL)_50_
(ME|EM)_50_	(GM|MG)_50_	(SI)_50_	(YD)_50_
(KK)_50_	(CC)_50_	(HS)_50_	(YG)_50_
(KM|MK)_50_	(MA|AM)_50_	(PP)_50_	(KK)_50_
(QH|HQ)_50_	(VV)_50_	(SH)_50_	(GY)_50_

Note: ^a.^ (bi-residue)_50_ means a 100-residue sequence with 50-time repeats of the indicated bi-residue. ^b.^ The character ‘|’ means ‘or’. The higher *R* value for T3_MM or SVM score for BPBAac, the more possibly being T3S effectors; vice versa.

Because there are too many combinations of amino acids in 100 positions, we cannot test all possible peptides with the method described above. A dynamic programming algorithm was further designed to find out the most favorable and unfavorable signal sequences for T3S recognition. The results were shown in Table 5. The continual stretch of proline was found to be most favorable. Interestingly, in the most unfavorable T3S sequence, proline covered nearly 1/3 of the total 100 positions (Table 5) further demonstrating the significant constraint imposed by adjacent amino acids in T3S sequences. The most favorable and unfavorable T3S sequences were also classified with BPBAac, with a confidently high and a low SVM score, respectively (Table 5).

**Table 5:** The most favorable and unfavorable T3S peptide sequence inferred by dynamic programming algorithm.

Property	Sequence	T3_MM_*R*_value	BPBAac_SVM
Most_favorable	PPPPPPPPPPPPPPPPPPPPPPPPPPPPPPPPPPPPPPPPPPPPPPPPPPPPPPPPPPPPPPPPPPPPPPPPPPPPPPPPPPPPPPPPPPPPPPPPPPPP	4.09	1.72
Least_favorable	YHWKPWKPWKPWKPWKPWKPWKPWPWKPWKPWKPWKPWKPWKPWKPWKPWKPWKPWKPWKPWKPWKPWKPWKPWKPWKPWKPWKPWPWKPWKPWKPWKPW	−4.97	−1.13

### Prediction and Comparison of *Salmonella* T3S Effectors

**Figure 5:**
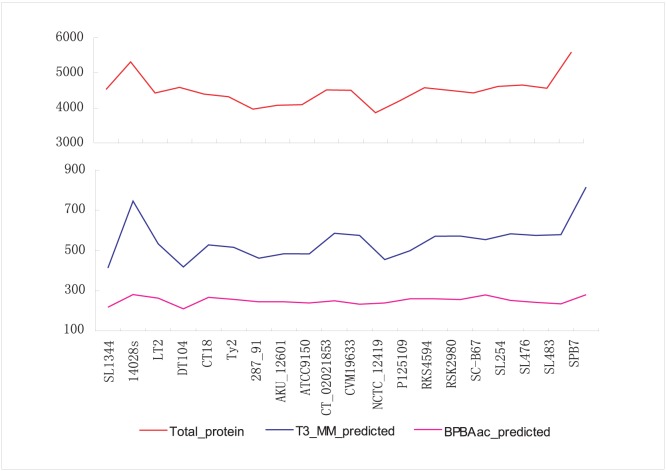
Summary of the total genome-encoding proteins, T3_MM predicted T3S effectors and BPBAac predicted T3S effectors in *Salmonella*. The total protein number for each *Salmonella* strain was depicted and linked with a line in red, while the number of T3S effectors predicted by T3_MM and BPBAac was shown in blue and purple, respectively. The patterns of these three lines were generally similar with moderate difference.

T3_MM was applied to predict T3S effectors from *Salmonella* genomes of different serovars (Methods). The number of predicted effectors was generally proportional to the genome size, with the number of T3_MM predicted effectors representing 12.2±1.3% of the total number of genome-encoding proteins. In comparison, the ratio of effector proteins predicted by BPBAac was 5.6±0.5% (Table S3 and S4; Fig. 5 and Table 6). A stable percentage of effectors (about 25% of the number predicted by BPBAac) were predicted by both software programs (Table 6; Table S5). For each strain, the number of effectors predicted by T3_MM was about 2-fold of that predicted with BPBAac (2.18±0.29; Table 6). Some recently identified effectors were successfully predicted by T3_MM but not BPBAac. For example, GtgE (i.e., SL14028 STM14_1196 and its orthologs in *S. Typhimurium* strains) was an effector validated recently, which could render *S. typhi* the ability to infect mice (Table S3 and S4) [23]–[24]. GtgE was not included in the training dataset of T3_MM or BPBAac, but it could be recalled by T3_MM rather than BPBAac. Therefore, though at the cost of more possible false positives, the larger number of candidates predicted by T3_MM should also provide more true T3S effectors.

**Table 6:** Summary of *Salmonella* effectors predicted by T3_MM and BPBAac.

Strain	Total_protein	T3_MM_predicted	BPBAac_predicted	Shared
SL1344	4527	413	218	51
14028s	5312	746	281	74
LT2	4425	533	263	70
DT104	4585	418	210	53
CT18	4395	528	267	71
Ty2	4318	516	257	68
287_91	3965	462	245	62
AKU_12601	4074	484	245	65
ATCC9150	4093	483	239	63
CT_02021853	4513	586	250	61
CVM19633	4501	575	233	60
NCTC_12419	3863	455	239	67
P125109	4206	499	260	65
RKS4594	4574	571	260	64
RSK2980	4500	572	256	64
SC-B67	4427	554	279	69
SL254	4613	583	252	64
SL476	4651	575	242	60
SL483	4562	579	235	57
SPB7	5591	815	280	67

The shared effectors predicted by both T3_MM and BPBAac were further analyzed since these effectors should have both position-specific and adjacent-constraint T3S signal features and therefore could be more likely to be true ones (Table S5). There were 70 effectors predicted by both programs in *S. enterica serovar* Typhimurium LT2 (Table 6); 17 (24.3%) of these were currently known T3S effectors (Table S5, Type I or II). Another 3 of the predicted genes, STM2584, STM1318 and STM2050, may also encode T3S effectors (Table S5, Type III), based on predictions taking into account various structural features (Wang *et al.*, unpublished data). 13 genes (18.6%, 13/70) were hypothetical with unknown function, or originated from bacteriophages (Table S5, Type IV). Two other genes, invE and STM1082, were closely related with T3SS function (Table S5, Type V). InvE encodes an accessory protein which is necessary for type 3 secretion of substrates while *STM1082* encodes an AraC-family transcriptional regulator, an important type of T3SS gene regulator [2]. It is worth noting that there were also predicted effectors (11.4%, 8/70), which were annotated as flagella-related proteins (Table S5, Type VI). T3SSs were reported to be evolutionarily related to flagella, and therefore, the flagella-related proteins and T3S effectors could share many sequence properties. These genes could also be improperly annotated as flagella-related proteins. The remaining 27 predicted effectors could participate in different biological processes or pathways (Table S5, Type VII).

The number of predicted effectors was different in different *Salmonella* strains (Table S5). It is interesting to identify strain-specific effectors. A comprehensive comparison was consequently performed to the effector sets in 3 *Salmonella* serovar Typhimurium strains, LT2, 14028s, and SL1344. The LT2 genome was sequenced ten years ago, while the genomes of 14028s and SL1344 were published recently [25]–[26]. Table 7 showed the specific effectors of individual strains. Two LT2-specific genes, STM2703 and STM0909, both encoding hypothetical proteins with unknown function, did not have orthologs in 14028s or SL1344 (Table 7, in bold). It is possible that STM2703 and STM0909 were obtained by horizontal transfer events. SL1344 and 14028s have two and four potential horizontally-acquired strain-specific genes, respectively, including known sopE (SL2674) in SL1344 (Table 7, in bold). In addition to the possible effectors without sequence homologs, each Typhimurium strain also had strain-specific effectors with sequence homologs in at least one other strain (Table 7, in italic). Closer analysis of the homologous sequences in other strains indicated they may have lost T3S signal features as a result of mutation and therefore were not recognized by T3SS.

**Table 7:** Predicted strain-specific T3S effectors.

LT2	SL1344	14028s
*STM2727*	**SL2674**	*STM14_4922*
*STM4377*	*SL1076*	**STM14_1479**
**STM2703**	**SL2715**	**STM14_2428**
**STM0909**	*SL4268*	**STM14_5051**
*STM4539*	*SL0536*	**STM14_1417**
*STM4202*	*SL3702*	*STM14_0039*
	*SL2941*	*STM14_0118*
	*SL0277*	*STM14_0489*
		*STM14_3042*
		*STM14_5206*

## Discussion

It is still an enigma how bacterial type III effectors are specifically recognized and secreted by type III secretion conduits. Previous experimental and bioinformatic analysis indicated that the signal sequences of type III effectors contained specific amino acid composition, such as being enriched in serine residues [10], [15], [17]. It is not yet known whether the amino acid composition influences the specificity of type III secretion, although some researchers have hypothesized that the amino acid composition is important for keeping the sequence more flexible and useful for specific recognition [7]. In this research, we further analyzed specific Aac features of T3S proteins. Our results highlighted the existence of Aac dependence relationship between adjacent positions in T3S signal sequences (Fig. 1). A closer observation and exploration of the dependence between adjacent amino acids could possibly provide interesting clues about the evolution and recognition mechanisms of type III signal sequences.

In this research, we used a simple Markov model-based variable for the Aac dependent on adjacent residues, followed by an observation and approximation of the variable distribution, for prediction of new T3S effectors [15], [17]–[20]. The Markov model was adopted because it is simple, statistically sound and can be well fit to time or space sequences. It also has been widely applied in biological sequence modeling. Our model was proved to be both effective and quantitative for predicting T3S effectors. The simplicity of the model suggests that further exploration of the Aac features of T3S signal sequences could yield important information about secretion recognition. Simplicity also ensures a more stable classification performance of the model, as was demonstrated by training data and different test datasets (Tables 1, 2, 3). We also tried other learning and classifying models, including widely adopted SVM, GLM and RF. However, none of these methods was comparable to T3_MM, to classify the T3S and non-T3S proteins based on the conditional Aac features (Table S1). This could be caused by the conditional Aac features, which are sequence-based and therefore more suitable for a sequence-based Markov model. The T3_MM model is sequence-based rather than position-based, therefore, the model is tolerant to insertion or deletion of one or several amino acids, or the possibility of alternative start codons (data not shown). In addition, the T3_MM model is not sensitive to the variance of sequence length because it has been normalized for sequence length. We also developed a wT3_MM model weighted by the probabilities of individual amino acids. Although the wT3_MM R values seemed better fit to known normal distributions, the classifying performance was not as good as that of original R values (Fig. 3 and Table 1). Therefore, we recommend that T3_MM be applied in practice instead of wT3_MM.

Effective T3 and BPBAac are two T3S effector prediction tools with the best prediction performance. The T3_MM model consistently outperformed Effective T3 with the training dataset and on test datasets from *Ralstonia* and *Pseudomonas* species (Table 2 and 3). BPBAac performed best for classification of the training dataset (Table 2), but significantly worse than T3_MM when new independent datasets were used, especially for sensitivity and accuracy (Table 3). Excellent inter-genera classification results further demonstrated the stable performance of T3_MM (Fig. 4). In practice, users are suggested to adopt different strategies to apply the software for different objectives. T3_MM is a better choice when more effectors are expected to be found, because T3_MM can predict more true effectors which other software cannot predict correctly (e.g., the effector GtgE in *S. Typhimurium*, Tables S3 and S4). This is especially useful for identification of new effectors in model species, in which many effectors have been found and highly-specific software, such as BPBAac, could not provide new candidates. To combine T3_MM and BPBAac is also a good choice when higher specificity is desired. The prediction results of this combinational strategy have both sequential bi-residue composition features and position-specific Aac features, and these candidates are more specific, though with a tradeoff being a loss of many new effectors. In this research, we first predicted *Salmonella* T3S effectors with T3_MM, and found it could give a larger number of possible candidates, some of which were recently validated by experiments and not predicted by BPBAac. A combined prediction strategy was further used to screen more specific effectors for further comparison and analysis (the percentage of known effectors in all predictions was increased to ∼25% from ∼5% for T3_MM and ∼9% for BPBAac). Many candidates were predicted by both programs, and most of them were conserved among different *Salmonella* serovars and stains (Table S5). In addition to the conserved and well-characterized effectors, the strain-specific ones, especially those whose encoding genes reside in a mobile region (e.g., prophage), are more interesting (Table 7). These potential effectors could be obtained through strain-specific horizontal gene transfer events, and exert a strain-specific molecular function. Careful comparison of the effectors among different bacterial strains would give some clues about the evolution or other knowledge of T3S effectors. For example, *Salmonella* was diverged to two currently observable species, *S. enterica* and *S. bongori*. All *S. enterica* strains contain two T3SSs (SPI-1 and SPI-2) while *S. bongori* didn’t obtain the SPI-2 T3SS [27]. Comparison of the *S. bongori* predictions with those of *S. enterica* strains, indicated that none of known SPI-2 T3S effectors were present while most SPI-1 effectors were present in *S. bongori.* This indicates that SPI-1 effectors were likely acquired before divergence of *Salmonella* species and that SPI-2 effectors may have been acquired in *S. enterica* subspecies or strains after the species divergence (Table S5; data not shown). The effector SlrP, which was previously considered as an effector of both SPI-1 and SPI-2 T3SSs [28], was present in *S. bongori*. This is an indication that *slrP*, as well as other inter-species conserved candidate effector genes, could participate in the SPI-1 T3SS activities. There were also many predicted *S. bongori* effectors that were not identified in *S. enterica*. These predicted SPI-1 T3SS effectors could potentially play important, species-specific and phenotype-specific roles in *S. bongori* and its interaction with cold-blooded hosts [27].

Based on the likelihood ratio matrices of adjacent bi-residues (and the first-position residues) between T3S and non-T3S proteins, the most favored and unfavored T3S sequence compositions were calculated using a dynamic programming algorithm (Table 5). The most preferred sequence was not composed of a stretch of serine resiudes, as was expected. This was also demonstrated by modelling analysis (Table 4). The results indicate that, due to the constraint exerted by neighbouring positions, the most significant preference of some residue (eg., serine) doesn’t necessarily mean the similar preference of continuous composition of that residue. In simulating analysis, however, we found a stretch of proline was most favorable, though proline was not as preferred by T3S proteins as serine in most N-terminal positions (Table 4 and 5). Experiments are required to test whether this is the case. Interestingly, the proline was also present in about 1/3 of the positions in the most unfavorable T3S sequence, further indicating the constraint of amino acid composition cause by its adjacent position (Table 5). It should be pointed out that in this modeling process, position information (i.e., position-specific bi-residue composition) was not considered, which could be more accurate theoretically but requires more known T3S proteins. Based on currently available validated T3S effectors, we calculated the most favorable T3S sequence by dynamic programming algorithms. The results were far different (e.g., the most favorable sequence of N-terminal 10-aa peptide being ‘TSWFAGDEKK’), and yet they were not stable, especially for the residues close to the C-termini. As more T3S effectors are validated, modeling based on position-specific bi-residue composition will become more feasible. These types of models are more likely to reveal hidden T3S motifs or unique amino acid composition features.

In conclusion, the T3_MM model that we have developed can be applied to identify new effectors from different bacterial species based on their genome sequences. As more T3S proteins are experimentally identified, the estimated parameters can be optimized and the model can be improved.

## Methods

### Data Source

The T3S and non-T3S training protein dataset was described in detail previously (Text S1) [17]. The dataset consisted of 154 non-homologous validated T3S proteins and 308 non-T3S proteins. The proteins were manually collected from different animal and plant pathogens or symbiotic bacteria, except *Ralstonia* [2], [17]. The non-T3S proteins were selected from the proteins encoded by different bacteria strains after removing the known effectors.

Two independent datasets were used for testing and comparing the performance of T3_MM and other software programs. For the first test dataset, the *Ralstonia* validated effectors were collected from a recent large-scale experimental study [21]. These effectors were identified by Cya translocation assay [21]. In total, 35 effectors and randomly selected 70 non-effectors from *Ralstonia* were included in this dataset (Text S2). None of the *Ralstonia* proteins were used in training dataset. The other testing dataset consists the most comprehensive list of known *Pseudomonas* effectors (Text S3) [22]. These effectors were annotated from literature with different experimental evidence [22]. 291 known effectors and 582 randomly selected control proteins from *Pseudomonas* were included. The control proteins were selected with a strategy similar with that of the training dataset. The ratio of size between positive and negative training/testing sequences was maintained at 1∶2, according to previous experience and related reports [17], [29].

### Assumption, Definition and Markov Model

Let vector 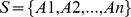 denotes a peptide sequence in which *A* represents amino acid while the number represents position and n represents the total length of *S*. Besides, let *A*_0_ denotes a hypothetically initial state of sequence *S*. Assume *A*_0_ probability *P*(*A*_0_) is 1 for each sequence *S*. The conditional probability of *A*_i+1_ on the amino acid at sequentially preceding position is denoted as: *P*(*A*_i+1_|*A*_i_), where 0≤*i*<n. Assume the amino acid composition at one position is only dependent on its sequentially preceding position; consequently, the generating probability of an sequence *S* could be described as an one-order Markov chain:
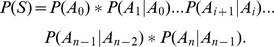



Assume there are two categories of sequences (*C*_1_ and *C*_2_), and S could be sampled either from *C*_1_ or *C*_2_. The likelihood of sequence S belonging to category *C* is:
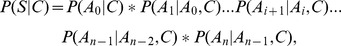
where *C* represents *C*_1_ or *C*_2_.

For each sequence *S*, a statistic variable *R*, is constructed to describe logarithm of the likelihood ratio between *P*(*S*|*C*_1_) and *P*(*S*|*C*_2_), i.e.,
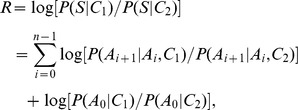
where the base is set as 2, and log[*P*(*A*_i+1_|*A*_i_,*C*_1_)/*P*(*A*_i+1_|*A*_i_,*C*_2_)] is predefined as zero when *P*(*A*_i+1_|*A*_i_,*C*) equals to 0.

For the models studied in this work, *C*_1_ and *C*_2_ represent T3S and non-T3S proteins, respectively. The probabilities of individual amino acids for T3S and non-T3S proteins, are used as the coefficients for weighted T3_MM model (wT3_MM).

T3_iAA assumes that amino acids in sequence *S* are independent on each other, and the probabilities of different *S* with fixed length of n sum to 1. Therefore, the probability of *S* equals to the probability product of each constitute amino acid. T3_iAA also calculates the logarithm value of likelihood ratio that *S* being T3S or non-T3S protein.

All the conditional probabilities, *P*(*A*_i_|*C*) or *P*(*A*_i+1_|*A*_i_,*C*) (0≤*i*<n, *C* = *C*_1_ or *C*_2_), are estimated using maximum likelihood method. The negative logarithm of probability is also calculated for more direct comparison and observation.

### Probability Distribution, Parameter Estimation and Decision Function

The *R* values for each T3S protein are calculated, and a histogram is drawn afterwards to represent the *R* distribution. A density curve is derived from the normal Q–Q plot and Shapiro-Wilk normality test are further adopted to test whether the observed distribution is a normal distribution. For normal distribution, the parameters, mean (μ) and standard deviation (σ), are estimated using a maximum likelihood method. The *R* values for non-T3S proteins, and weighted *R* values for T3S and non-T3S proteins, are all calculated and analysed with the same strategy.

*R* values of T3S and non-T3S proteins are ideally fitting to two distinct probability distributions: *F*_C1_ and *F*_C2_, respectively. Suppose mean μ(*F*_C1_) is not smaller than μ(*F*_C2_). For any sequence S and its *R* value *r*, *P*_C1_(*R*|*R≤r*) and *P*_C2_(*R*|*R≥r*) represent the probability of *S* according to *F*_C1_ and *F*_C2_ distribution, respectively. Let discriminant function D(*r*) = *P*_C1_(*R*|*R≤r*)−*P*_C2_(*R*|*R≥r*). If D(*r*)≥0, *r* follows *F*_C1_ distribution and *S* belongs to *C*_1_ or T3S proteins; otherwise, D(*r*)<0, r follows *F*_C2_ distribution and *S* belongs to *C*_2_. When *r* follows *F*_C1_ distribution, the probability of S being a *C*_1_ or T3S protein is the mean of *P*_C1_(*R*|*R≤r*) and 1−*P*_C2_(*R*|*R≥r*). When *r* follows *F*_C2_ distribution, the probability of S being a *C*_2_ or non-T3S protein is the mean of *P*_C2_(*R*|*R≥r*) and 1−*P*_C1_(*R*|*R≤r*).

### Performance Assessment

Accuracy (*A*), Specificity (*Sp*), Sensitivity (*Sn*), Receiver Operating Characteristic (*ROC*) curve and Matthews Correlation Coefficient (*MCC*) were utilized to assess the predictive performance. In the following formula, *A* denotes the percentage of both positive instances (T3S) and negative instances (non-T3S) correctly predicted. *Sn* (true positive rate) and *Sp* (true negative rate) respectively represent the percentage of positive instances (T3S) and the percentage of negative instances (non-T3S) correctly predicted. An *ROC* curve is a plot of Sn versus (1-*Sp*), and is generated by shifting the decision threshold. *AUC* gives a measure of classifier performance. *MCC* takes into account true and false positives and false negatives and is generally regarded as a balanced measure which can be used even if the classes are of very different sizes.
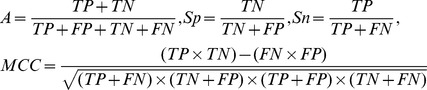
where *TP*, *TN*, *FP* and *FN* denotes the number of true positives, true negatives, false positives and false negatives, respectively.

To avoid overfitting of T3_MM, a 5-fold cross-validation strategy was adopted to evaluate its classification performance. The training dataset was divided into 5 subsets, each with identical number of positive and negative sequences. Four subsets were put together for training and the remaining subset was used for testing. The process was repeated so that all sequences were tested once. The performance parameters were evaluated as the average values of the cross-validation results.

To test the robustness of T3_MM, an inter-species cross-validation strategy was adopted. The T3S and non-T3S proteins of a targeted genus (or phylum/subgroup) were extracted from the training dataset at first. The remaining sequences were used to re-train the model, which in turn was used to test the proteins of targeted genus (or phylum/subgroup). The sensitivity and specificity were calculated thereafter.

### Comparison with other Software Programs

The Support Vector Machine (SVM), Generalized Linear Model (GLM) and RandomForest (RF) were also used for training the conditional Aac features. Because the size of features was large (400), and the length of signal sequences was 100 aa, only the bi-residues statistically different between T3S and non-T3S sequences were included for training (binomial test, *FDR*-corrected p<0.05). R packages were adopted to implement SVM (‘e1071’), GLM (‘faraway’) and RF (‘RandomForest’), respectively (http://cran.r-project.org/).

The performance of T3_MM was also compared with other established T3S protein prediction models, Effective T3 [15] and BPBAac [17]. The original methods and optimal parameters were used for re-training the models with new training datasets. To compare the performance of each type of software to classify the two testing datasets, the default parameters were used, except sensitive cutoff values were adopted for either model (a probability of 0.95 for Effective T3 and an SVM value of 0.0 for BPBAac), since selective cutoff values for both models gave quite low recalls for the test datasets [15], [17].

### Most and Least Possible T3S Signal Sequences

A dynamic programming algorithm was designed to find out the most and least possible T3S signal sequences based on the log odds of probabilities of individual amino acids and di-residues. Local maximal (minimal) sum of log odds of first-position individual amino acids and sequential di-residues between T3S and non-T3S sequences were recursively calculated. The path for continual local maximal (minimal) values was recorded and it was recognized as the most (least) possible T3S signal sequence.

### Prediction, Comparison and Annotation of *Salmonella* Effectors

Genome and genome-encoding protein sequences of Salmonella strains were downloaded from the NCBI website: http://www.ncbi.nlm.nih.gov/genome. The strains and their genome accessions included: *S. typhimurium* LT2 (NC_003197), 14028s (CP001363), SL1344 (NC_016810), DT104, *S. typhi* CT18 (NC_003198), Ty2 (NC_004631), *S. paratyphi* A AKU_12601 (NC_011147), ATCC9150 (NC_006511), *S. paratyphi* B SPB7 (NC_010102), *S. paratyphi* C RKS4594 (NC_012125), *S. enteritidis* P125109 (AM933172), *S. gallinarum* 287/91 (NC_011274), *S. dublin* CT_02021853 (NC_011205), *S. Schwarzengrund* CVM19633 (NC_011094), *S. newport* SL254 (NC_011080), *S. heidelberg* SL476 (NC_011083), *S. choleraesuis* SC-B67 (NC_006905), *S. agona* SL483 (NC_011149), *S. bongori* NCTC_12419 (NC_015761), and *S. arizonae* RSK2980 (NC_010067). The genome-encoding sequences were input to the T3_MM server (http://biocomputer.bio.cuhk.edu.hk/T3_MM.php) with default parameters for T3S protein prediction. BPBAac (http://biocomputer.bio.cuhk.edu.hk/BPBAac.php) was also used to predict effectors in different bacteria. A sensitive cutoff value, 0.0, was set for BPBAac prediction. To find out the orthologs of two strains, a reciprocal alignment was performed to the protein sequences of any pair of genomes with blast program (blastp, evalue <0.0005). The orthologs were defined as the mutual best alignment hits. Gene order information was also analyzed and used as additional evidence. The Genbank annotations for LT2 and other genomes were referred for function annotation of predicted effectors.

## Supporting Information

Figure S1 **Amino acid composition difference between T3S and non-T3S sequences.** Horizontal axis: twenty types of amino acids. Vertical axis: negative logarithm of the composition probability of corresponding amino acid.
(PDF)

Figure S2 **Comparison of Aac probability profiles conditional on preceding-position amino acid.** Horizontal axis: sequentially adjacent two amino acids. Vertical axis: negative logarithm of the conditional probability of corresponding bi-amino acids (bi-aa). T3S and non-T3S sequences were shown in black bars and grey bars, repectively. WW, WY and MV were not present in T3S sequences, therefore, the probability for these residues was replaced with 1/1000 so as to avoid an infinite logarithm value. Bi-aa with conditional probability significantly different from absolute probability in T3S sequences but not significant in non-T3S sequences were marked with a star above the bar (T test, *p*<0.05). Among bi-aas with the same first-position residue in T3S sequences, bi-aa with the rank of conditional probability significantly different from that of absolute probability was marked with an upward (rank difference between conditional and absolute probability ≤−5; the smaller the rank value, the higher the probability) or downward arrow (difference ≥5) above the bar. Similarly, among bi-aas with the same first-position residue, bi-aa with the rank of conditional probability in T3S sequences significantly different from that of non-T3S sequences was marked with a backward arrow (rank difference between conditional probability of T3S and non-T3S sequences ≤−5) or forward arrow (difference ≥5) below the bar.
(PDF)

Table S1 **Performance comparison for Markov model (T3_MM), SVM, GLM and RF training the conditional Aac features.**
(DOC)

Table S2 **Predicted score of simulated sequences.**
(XLS)

Table S3 **T3_MM predicted** ***Salmonella*** **effectors.**
(XLS)

Table S4 **BPBAac predicted** ***Salmonella*** **effectors.**
(XLS)

Table S5 ***Salmonella*** **effectors predicted by T3_MM and BPBAac.**
(XLS)

Text S1 **Training sequences.**
(TXT)

Text S2 **Mukaihara 2010** ***Ralstonia*** **T3S proteins.**
(TXT)

Text S3 **Baltrus 2011 comprehensive** ***Pseudomonas*** **T3S protein.**
(TXT)
