# Supplementary figures and images for: T3_MM: A Markov Model Effectively Classifies Bacterial Type III Secretion Signals
Source: PLoS One. 2013 Mar 5;8(3):e58173. doi: 10.1371/journal.pone.0058173 (PMC3589343; doi:10.1371/journal.pone.0058173)

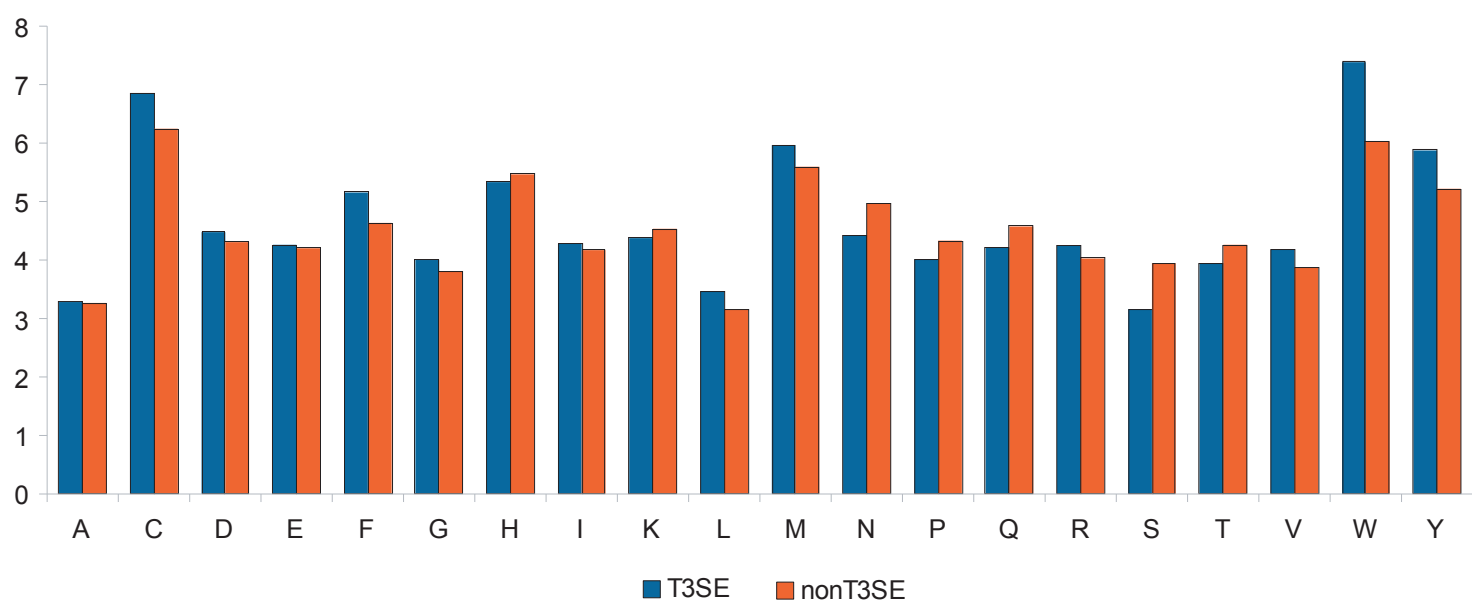

Supplement: Figure S1 — Amino acid composition difference between T3S and non-T3S sequences. Horizontal axis: twenty types of amino acids. Vertical axis: negative logarithm of the composition probability of corresponding amino acid. (PDF) [file pone.0058173.s001.pdf]

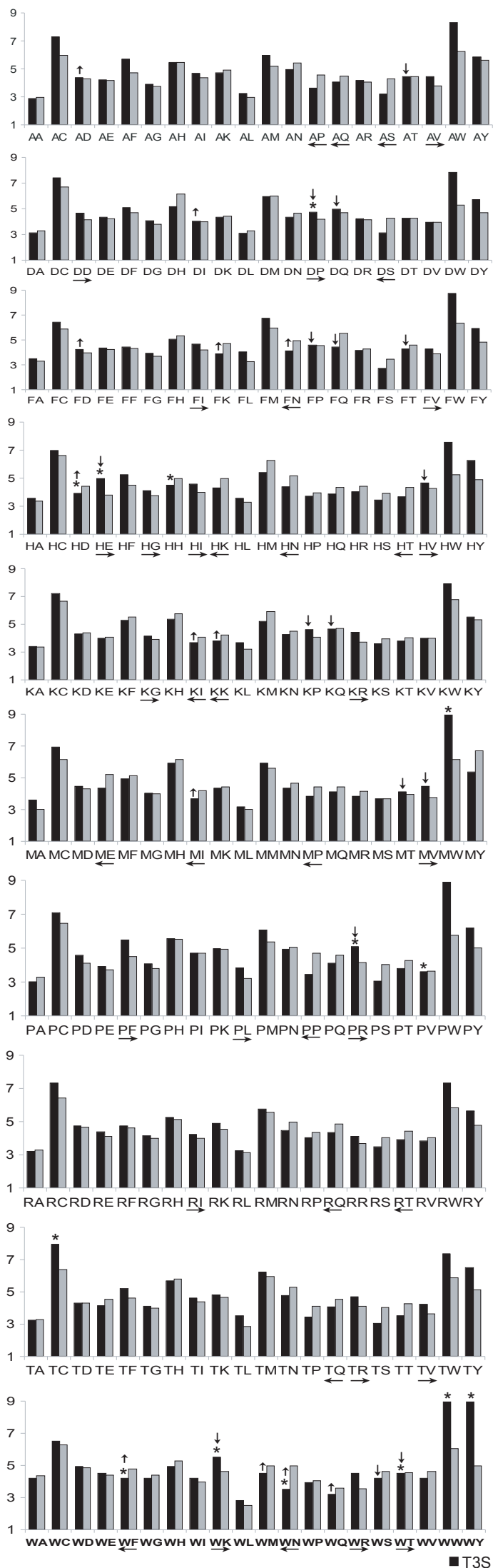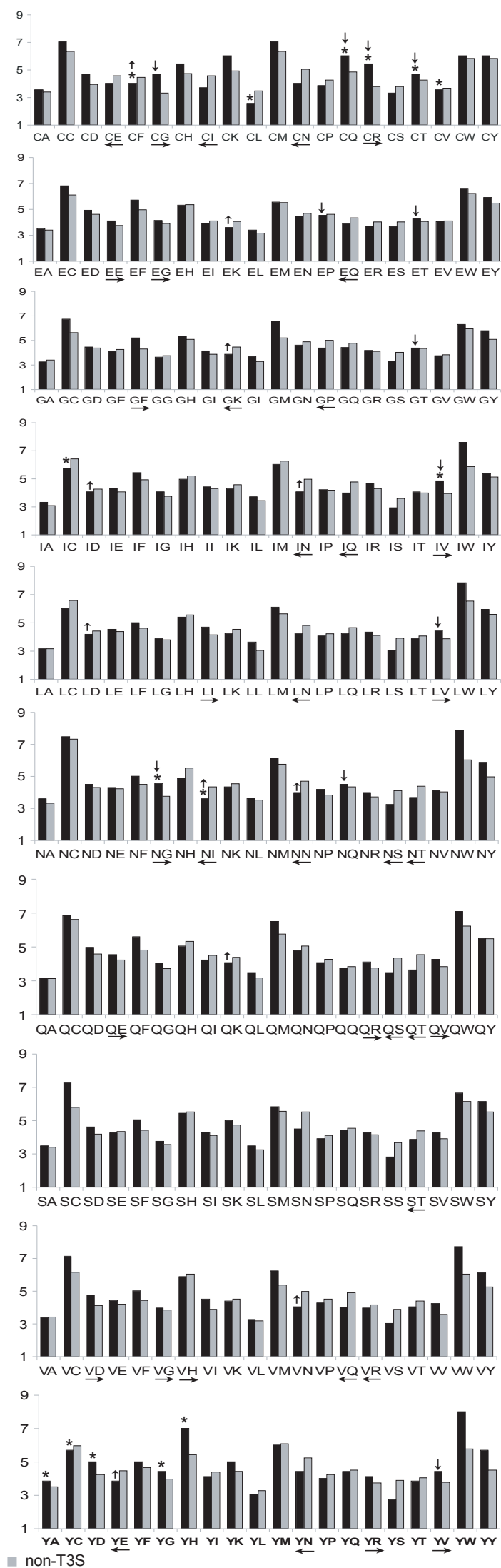

Supplement: Figure S2 — Comparison of Aac probability profiles conditional on preceding-position amino acid. Horizontal axis: sequentially adjacent two amino acids. Vertical axis: negative logarithm of the conditional probability of corresponding bi-amino acids (bi-aa). T3S and non-T3S sequences were shown in black bars and grey bars, repectively. WW, WY and MV were not present in T3S sequences, therefore, the probability for these residues was replaced with 1/1000 so as to avoid an infinite logarithm value. Bi-aa with conditional probability significantly different from absolute probability in T3S sequences but not significant in non-T3S sequences were marked with a star above the bar (T test, p<0.05). Among bi-aas with the same first-position residue in T3S sequences, bi-aa with the rank of conditional probability significantly different from that of absolute probability was marked with an upward (rank difference between conditional and absolute probability ≤−5; the smaller the rank value, the higher the probability) or downward arrow (difference ≥5) above the bar. Similarly, among bi-aas with the same first-position residue, bi-aa with the rank of conditional probability in T3S sequences significantly different from that of non-T3S sequences was marked with a backward arrow (rank difference between conditional probability of T3S and non-T3S sequences ≤−5) or forward arrow (difference ≥5) below the bar. (PDF) [file pone.0058173.s002.pdf]
