# Supplementary material for: T3_MM: A Markov Model Effectively Classifies Bacterial Type III Secretion Signals
Source: PLoS One. 2013 Mar 5;8(3):e58173. doi: 10.1371/journal.pone.0058173 (PMC3589343; doi:10.1371/journal.pone.0058173)
Supplement: Table S1 — Performance comparison for Markov model (T3_MM), SVM, GLM and RF training the conditional Aac features. (DOC) [file pone.0058173.s003.doc]

**Table S1. Performance comparison for Markov model (T3_MM), SVM, GLM and RF training the conditional Aac features.** The parameters were evaluated based on a 5-fold cross-validation strategy. The standard deviations for *Sn*, *Sp* and *A* were also indicated.

| **Software** | ***S_n_* (%)** | ***S_p_* (%)** | ***A* (%)** | **MCC** |
| --- | --- | --- | --- | --- |
| T3_MM | 83.87 (±5.10) | 90.32 (±5.93) | 88.17 (±3.10) | 0.7362 |
| SVM | 58.71 (±5.30) | 92.58 (±4.36) | 80.65 (±2.28) | 0.5029 |
| GLM | 55.48 (±4.78) | 72.25 (±5.97) | 66.67 (±5.21) | 0.3837 |
| RF | 58.06 (±6.45) | 81.94 (±5.52) | 73.98 (±4.89) | 0.4370 |
